# VPAC2 receptor expression in human normal and neoplastic tissues: evaluation of the novel MAB SP235

**DOI:** 10.1530/EC-14-0051

**Published:** 2015-01-07

**Authors:** Stefan Schulz, Anika Mann, Benjamin Novakhov, Hugh D Piggins, Amelie Lupp

**Affiliations:** Institute of Pharmacology and Toxicology, Jena University Hospital, Friedrich Schiller University Jena, Drackendorfer Straße 1D-07747, Jena, Germany; 1 Faculty of Life Sciences, University of Manchester, Manchester, M13 9PT, UK

**Keywords:** VIP/PACAP receptors, antibodies, immunocytochemistry, western blot, immunohistochemistry

## Abstract

The vasoactive intestinal peptide receptor 2 (VPAC2) is widely distributed throughout the body and is also overexpressed in a variety of human neoplastic tissues. However, little is known about its precise tissue distribution, regulation and function, which is in part be due to the lack of specific monoclonal anti-VPAC2 antibodies. In this study, we extensively characterised the novel rabbit monoclonal anti-VPAC2 antibody (clone SP235) using transfected cells and mouse, rat and human tissues. SP235 was then subjected to a comparative immunohistochemical study on a series of 167 histological specimens from formalin-fixed, paraffin-embedded human tumours and adjacent normal tissues. SP235 detected a broad band migrating at a molecular weight of 50–70 kDa in western blotting analyses of various mouse tissues as well as VPAC2- but not VPAC1-transfected human embryonic kidney 293 cells. SP235 yielded an efficient immunostaining of distinct cell populations in human tissue samples with a predominance of plasma membrane staining, which was completely abolished by preadsorption with its immunising peptide. SP235 immunohistochemistry detected VPAC2 receptors in lymphocytes present in spleen, tonsils, lymph nodes and Peyer's patches, chief cells of gastric mucosa, exocrine and endocrine pancreas, kidney tubules and blood vessels. In addition, VPAC2 was observed in thyroid, gastric and lung carcinomas, pancreatic adenocarcinomas, sarcomas and neuroendocrine tumours. SP235 may prove of great value in the identification of VPAC2 receptors during routine histopathological examination. VPAC2 visualisation with this simple and rapid immunohistochemical method will facilitate identification of candidate tumours for vasoactive intestinal peptide (VIP)-based diagnostics or therapeutic interventions.

## Introduction

The overexpression of receptors for small regulatory peptides, e.g. somatostatin, bombesin, vasoactive intestinal peptide (VIP) or pituitary adenylate cyclase-activating peptide (PACAP) provides the opportunity for effective clinical application of a variety of peptide analogues in the diagnosis and treatment of human tumours. VIP is a 28-amino acid neuropeptide, which is widely distributed throughout the brain and periphery (1). The biological actions of VIP are mediated by a family of three G protein-coupled receptors designated vasoactive intestinal peptide receptor 1 (VPAC1), VPAC2 and pituitary adenylate cyclase-activating peptide receptor 1 (PAC1). The PAC1 receptor exhibits lower affinity for VIP than for PACAP, while VPAC1 and VPAC2 receptors exhibit similar affinities for VIP and PACAP (1, 2, 3). Earlier, mRNA, Western blotting and immunohistochemical studies have shown that VIP/PACAP receptors are expressed in the great majority of the most frequently occurring human tumours, including breast, ovarian, prostate, pancreas and colon carcinomas, insulinoma, carcinoid, glioblastoma, meningioma, pituitary adenoma and pheochromocytoma (4, 5, 6, 7, 8, 9, 10, 11, 12, 13, 14). More recent studies have shown that VIP/PACAP receptor-positive mammary, intestinal and endocrine tumours can be visualised by *in vivo* VIP receptor scintigraphy (11, 15, 16, 17, 18, 19, 20, 21, 22, 23, 24, 25).

To evaluate whether a patient is a candidate for *in vivo* VIP receptor targeting, it is of great advantage to know the receptor expression of the tumour. However, the widespread application of immunohistochemistry for *in vitro* VIP receptor evaluation has been hampered by the lack of MABs and the limited availability of specific polyclonal antibodies. Recently, we have extensively characterised three novel rabbit MABs against the sst2A receptor, the sst5 receptor and the sst3 receptor named UMB1, UMB4 and UMB5. We have shown that these antibodies selectively detect their cognate receptor in crude membrane extracts from sst receptor-expressing cells and tissues and that they are excellently suited for the assessment of sst2A, sst5 or sst3 receptor expression in fixed human tissue samples (26, 27, 28). In this study, we demonstrate that the new rabbit monoclonal anti-VPAC2 antibody SP235 selectively detects its cognate receptor in formalin-fixed and paraffin-embedded tissues. Given the numerous advantages of rabbit MABs compared with currently available polyclonal antisera, the development of SP235 will facilitate the establishment of routine performance of VPAC2 immunohistochemistry in human tumours.

## Materials and methods

### Tumour samples and tissue preparation

All tissue specimens had been fixed in formalin and embedded in paraffin. The following tumours were investigated: gastric cancer (*n*=20); gastrointestinal stromal tumours (*n*=4); ductal pancreatic adenocarcinoma (*n*=10); small-cell lung carcinoma (*n*=5); lung adenocarcinoma (*n*=5); squamous cell carcinoma of the lung (*n*=3); sarcoma ((*n*=19); pleomorphic sarcoma (*n*=2), leiomyosarcoma (*n*=4), rhabdomyosarcoma (*n*=3), liposarcoma (*n*=4), angiosarcoma (*n*=2) and osteosarcoma (*n*=4)); thyroid carcinoma (*n*=2); breast carcinoma (*n*=10); ovarian carcinoma (*n*=12); cervical carcinoma (*n*=13); urinary bladder carcinoma (*n*=8); prostate carcinoma (*n*=7); neuroendocrine tumours (*n*=23); growth hormone (GH)-producing pituitary adenoma (*n*=21) and pheochromocytoma (*n*=5). Several of the tumours contained adjacent normal tissue, which was also analysed. In addition, tumour-free human tissue samples from lung, heart, liver, gallbladder, different parts of the gastrointestinal tract, pancreas, kidney, spleen, tonsils, thymus and lymph nodes (*n*=6 each) were also evaluated and the staining patterns compared with those seen in the tissues surrounding the tumours. In no case differences were noted.

### Plasmids

Plasmids encoding the full-length human VPAC1 receptor (NM_004624) or the human VPAC2 receptor (NM_003382) genes were obtained from the Missouri S&T cDNA Resource Center (Rolla, MO, USA).

### Antibodies

The rabbit monoclonal anti-VPAC1 antibody (SP234) and the rabbit monoclonal anti-VPAC2 antibody (SP235) were obtained from Spring Bioscience (Pleasanton, CA, USA). The identity of the immunising peptide for SP234 was TRVSPGARRSSSFQAEVSLV, which corresponds to amino acids 438–457 of the human VPAC1 receptor. The identity of the immunising peptide for SP235 was LQFHRGSRAQSFLQTETSVI, which corresponds to amino acids 419–428 of the human VPAC2 receptor. Given the high degree of sequence homology in the carboxyl-terminal domain of human, mouse and rat VPAC2 receptors, cross-reactivity of SP235 between human and rodent VPAC2 receptors is expected and detected.

### Cell culture and transfection

Human embryonic kidney 293 (HEK 293) cells obtained from the DSMZ (Braunschweig, Germany) were cultured at 37 °C and 5% CO_2_ in DMEM supplemented with 10% FCS. HEK 293 cells were stably transfected with either VPAC1 receptor or VPAC2 receptor. Stably transfected cells were grown in medium supplemented with 1 μg/ml gentamicin.

### Immunocytochemistry

Stably transfected HEK 293 cells were grown on poly-l-lysine-coated coverslips overnight, fixed with 4% paraformaldehyde and 0.2% picric acid in phosphate buffer (pH 6.9) for 30 min at room temperature and washed several times with phosphate buffer. The specimens were permeabilised with Triton-X and then incubated with anti-VPAC1 (SP234) or anti-VPAC2 (SP235) antibodies at dilutions ranging from 1:50 to 1:1000 overnight at 4 °C. The cells were washed several times with phosphate buffer and then incubated with Alexa 488-conjugated secondary antibody for 2 h at room temperature, mounted and examined using a Zeiss LSM 510 Meta laser scanning confocal microscope.

### Western blotting analysis

Stably transfected HEK 293 cells were seeded onto poly-l-lysine-coated 60 mm dishes and grown to 80% confluence. The cells as well as freshly dissected tissues from WT (C57BL6) and VPAC2-deficient mice, including small and large intestine, gastric mucosa, brain, pancreas, spleen and testis, were lysed in detergent buffer (50 mM Tris–HCl pH 7.4, 150 mM NaCl, 5 mM EDTA, 10 mM NaF, 10 mM disodium pyrophosphate, 1% Nonidet P-40, 0.5% sodium deoxycholate and 0.1% SDS) in the presence of protease and phosphatase inhibitors Complete Mini and PhosSTOP (Roche Diagnostics). The receptors were enriched using wheat germ lectin agarose beads as described previously (29, 30). Proteins were eluted from beads using SDS sample buffer for 20 min at 45 °C. The samples were then subjected to 7.5% SDS–polyacrylamide gels, and blotted onto PVDF membranes. The membranes were incubated with the rabbit monoclonal anti-human VPAC1 antibody (SP234; dilution 1:500) or the rabbit monoclonal anti-human VPAC2 antibody (SP235; dilution 1:500), followed by incubation with peroxidase-conjugated secondary anti-rabbit antibody (Santa Cruz Biotechnology; dilution 1:5000) and ECL detection (Amersham). For adsorption controls, antibodies were preincubated with 10 μg/ml of their cognate peptides for 2 h at room temperature.

### Immunohistochemistry

Four μm paraffin sections were cut and floated onto positively charged slides and immunohistochemically stained as described previously (31). Briefly, sections were dewaxed, microwaved in 10 mM citric acid (pH 6.0) for 20 min at 600 W and subsequently incubated with the rabbit monoclonal anti-human anti-VPAC2 antibody (SP235; dilution 1:500) overnight at 4 °C. Detection of the primary antibody was performed using a biotinylated goat anti-rabbit IgG followed by an incubation with peroxidase-conjugated avidin (Vector ABC ‘Elite’ Kit, Vector Laboratories, Burlingame, CA, USA). Binding of the primary antibody was visualised using 3-amino-9-ethylcarbazole (25) in acetate buffer (BioGenex, San Ramon, CA, USA). The sections were then rinsed, counterstained with Mayer's haematoxylin and mounted in Vectamount mounting medium (Vector Laboratories). For immunohistochemical controls, SP235 was either omitted or adsorbed for 2 h at room temperature with 10 μg/ml of the peptide used for immunisations.

All immunohistochemical stainings were evaluated by two independent investigators. In the case of a discrepancy in the scoring between the two investigators, a final decision was achieved by consensus. The staining intensity was evaluated as follows: −, no; +, mild; ++, moderate and +++, strong staining. A tumour sample was considered positive if at least a moderate (++) staining of the plasma membrane was observed with 30% of the tumour cells.

## Results

### Characterisation of the VPAC2 receptor antibody SP235

**Figure 1:**
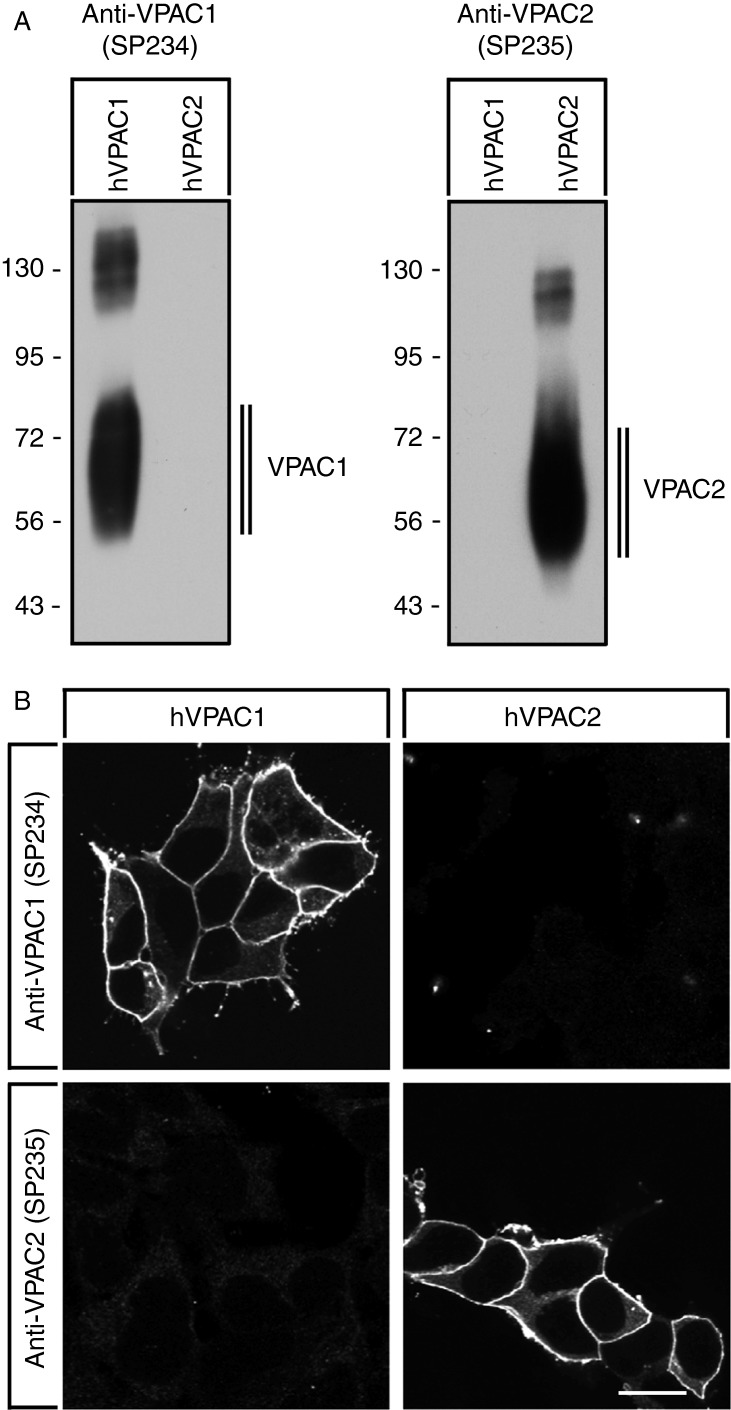
Characterisation of SP235 using transfected cells. (A) Western blotting analysis of the specificity of the anti-VPAC2 antibody SP235. Extracts from HEK 293 cells stably transfected to express either the human VPAC1 receptor (hVPAC1) or the human VPAC2 receptor (hVPAC2) were separated on 7.5% SDS–polyacrylamide gels and blotted onto PVDF membranes. Membranes were then incubated with the rabbit monoclonal anti-VPAC1 antibody (SP234) or with the rabbit monoclonal anti-VPAC2 antibody (SP235) at a dilution of 1:500. Blots were developed using ECL. Two additional experiments gave similar results. Ordinate, protein molecular weight marker (kDa). (B) Characterisation of SP235 by immunofluorescent staining of transfected cells. HEK 293 cells stably transfected to express either hVPAC1 or hVPAC2 were fixed and immunofluorescently stained with the anti-VPAC1 antibody (SP234) or the anti-VPAC2 antibody (SP235). Representative results from one of three independent experiments are shown. Scale bar, 20 μm.

**Figure 2:**
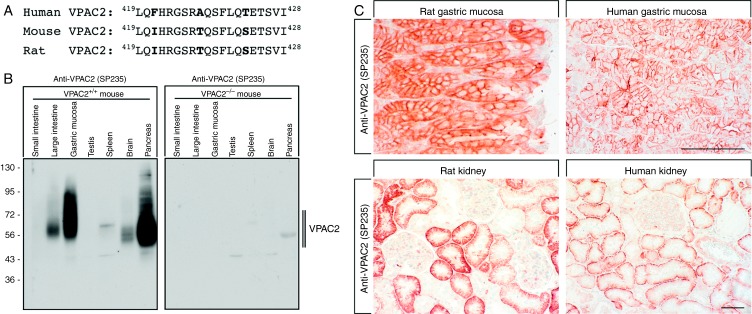
Characterisation of SP235 using mouse, rat and human tissues. (A) Comparision of the carboxyl-terminal sequences of mouse, rat and human VPAC2. The sequence depicted for the human VPAC2 was used for antibody generation. (B) Western blotting analysis of SP235 in various tissues. Tissue extracts from WT mice (*Vpac*^+^^/^^+^) and mice lacking *Vpac2* (*Vpac2*^−/−^) were separated on 7.5% SDS–polyacrylamide gels and blotted onto PVDF membranes. Membranes were then incubated with the rabbit monoclonal anti-VPAC2 antibody (SP235) at a dilution of 1:500. Membranes were developed using ECL. Ordinate, migration of protein molecular weight markers (kDa). (C) SP235 immunohistochemistry in rat and human tissues. Sections were dewaxed, microwaved in citric acid and incubated with the rabbit monoclonal anti-VPAC2 antibody (SP235) at a dilution of 1:500. Sections were then sequentially treated with biotinylated anti-rabbit IgG and AB solution. Sections were then developed in AEC and lightly counterstained with haematoxylin. Representative photomicrographs from one of five different tissue samples are shown. Scale bar: upper panel=200 μm and lower panel=100 μm.

**Figure 3:**
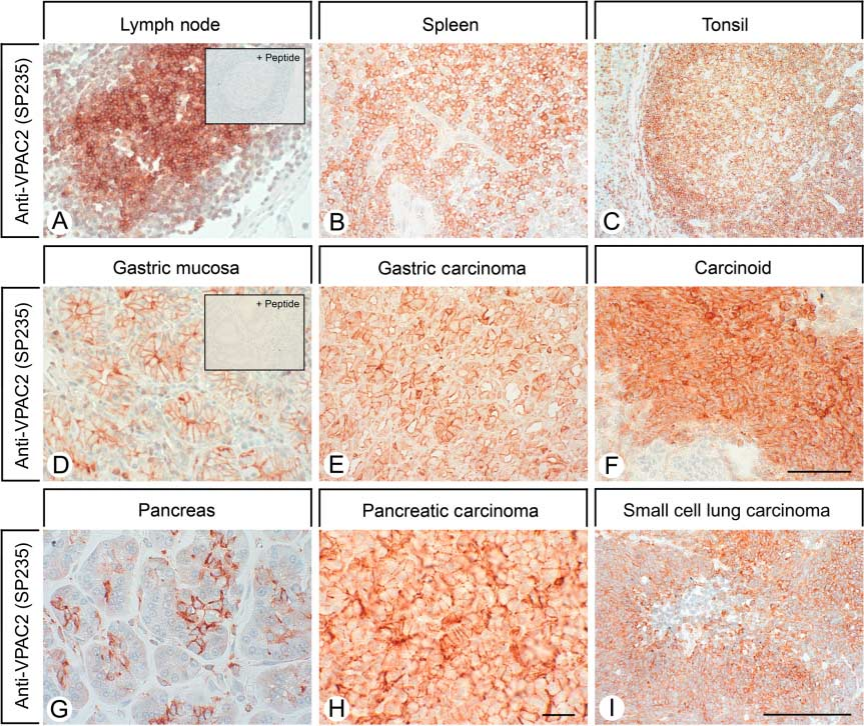
SP235 immunohistochemistry of human normal and neoplastic tissues. Sections were dewaxed, microwaved in citric acid and incubated with the rabbit monoclonal anti-VPAC2 antibody (SP235) at a dilution of 1:500. Sections were then sequentially treated with biotinylated anti-rabbit IgG and AB solution. Sections were then developed in AEC and lightly counterstained with haematoxylin. Insets in A and D, for adsorption controls the SP235 was incubated with 10 μg/ml of the peptide used for immunisations (+ peptide). Scale bar, A=B=C=F=I=250 μm and D=E=G=H=100 μm.

Specificity of the novel rabbit monoclonal anti-VPAC2 antibody (SP235) was monitored using western blotting analysis. When extracts from stably transfected HEK 293 cells were electrophoretically separated and blotted onto PVDF membranes, SP235 detected a broad band migrating at 50–70 kDa in cells stably expressing the VPAC2 but not in cells expressing the VPAC1 receptor (Fig. 1A, left panel). Conversely, the rabbit monoclonal anti-VPAC1 antibody (SP234) detected a broad band migrating at 55–75 kDa in cells stably expressing the VPAC1 receptor but not in cells expressing the VPAC2 receptor (Fig. 1A, right panel). SP235 was further characterised using immunofluorescent staining of transfected cells. As depicted in Fig. 1B, the anti-VPAC2 antibody (SP235) revealed prominent immunofluorescence localised at the plasma membrane only in VPAC2- but not in VPAC1-expressing cells. Conversely, the anti-VPAC1 antibody (SP234) revealed prominent immunofluorescence localised at the plasma membrane only in VPAC1, but not in VPAC2-expressing cells (Fig. 1B). Next, SP235 was tested for possible cross-reactivity with other proteins present in the extracts from mouse, rat or human tissues. A comparison of the carboxyl-terminal sequences revealed a high degree of homology between mouse, rat and human VPAC2 receptors (Fig. 2A). Consequently, cross-reactivity of SP235 to both mouse and rat VPAC2 receptors was observed (Fig. 2B and C). When extracts from a variety of mouse tissues were electrophoretically separated and blotted onto PVDF membranes, the anti-VPAC2 antibody (SP235) revealed a broad receptor-like band with a molecular weight similar to that observed for recombinant VPAC2 receptor in gastric mucosa, pancreas and brain from WT (C57BL6) but not from *Vpac2*-knock-out mice (Fig. 2B). In addition, SP235 revealed faint bands of similar size in large intestine and spleen, but not in small intestine or testis (Fig. 2B). Immunoreactive bands for SP235 were completely abolished by preabsorbtion with 10 μg/ml of its immunising peptide (Supplementary Figure S1, see section on supplementary data given at the end of this article). The anti-VPAC2 antibody (SP235) was then subjected to immunohistochemical staining of a selection of mouse, rat and human tissues revealing similar cellular and subcellular localisations of VPAC2 receptors in all three species. As depicted in Fig. 2C, VPAC2 receptors were detected at the plasma membrane of chief cells in the gastric mucosa and the basal membrane of proximal kidney tubules of both rats and humans. In addition, tissue immunostaining of SP235 was completely abolished by preadsorption with its immunising peptide (Fig. 3A and D).

### SP235 immunohistochemistry in normal and neoplastic human tissues

Initial experiments showed that heat-induced epitope retrieval is required for efficient SP235 immunohistochemical staining of human formalin-fixed and paraffin-embedded tissue (not shown). First, we analysed a variety of human normal tissues using SP235. Prominent immunostaining localised at the plasma membrane was observed in distinct populations of lymphocytes present in all major lymphatic organs, including germinal centres of lymph node follicles, white pulp of the spleen, lymphoid nodules of tonsils and follicles of Peyer's Patches (Fig. 3A, B and C). VPAC2 receptor immunoreactivity was also abundant at the plasma membrane of chief cells of gastric mucosa as well as at the apical membrane of cells in the exocrine pancreas (Fig. 3D and G). In mouse pancreatic islets, VPAC2 receptors were detected in all cells and in considerably higher densities than that in human and rat islets (not shown). In the kidney, SP235 immunohistochemistry revealed a discrete staining of the basal membrane of proximal tubules but not of glomeruli, distal tubules or collecting ducts (Fig. 2C). In addition, VPAC2 receptor immunoreactivity was also seen in the epithelium lining the colloid of the thyroid gland, in the Kupffer cells of the liver, in the smooth muscles of blood vessels and in the smooth muscle layers of the gastrointestinal tract, of the gallbladder and of the urinary bladder (Supplementary Figure S2, see section on supplementary data given at the end of this article). The rabbit monoclonal anti-VPAC2 antibody (SP235) was then subjected to immunohistochemical stainings of a series of 167 human tumours. All tumour samples were evaluated using a four-step scoring system as described in ‘Materials and methods’ section. A tumour sample was only considered positive if at least 30% of tumour cells exhibited a moderate staining of the plasma membrane. The results are summarised in Table 1. A high prevalence of VPAC2 receptors was detected in some of the most frequently occurring carcinomas, including gastric cancer, pancreatic ductal adenocarcinoma, small-cell lung carcinoma and thyroid carcinoma. SP235 immunohistochemistry revealed VPAC2 receptor expression also in a proportion of breast, ovarian and cervical carcinomas. In contrast, VPAC2 receptors were not observed in urinary bladder or prostate carcinomas. In addition, SP235 detected a high number of positive cases in neuroendocrine tumours of the gastrointestinal tract and GH-producing pituitary adenomas, but not in pheochromocytomas (Supplementary Figure S2). In most of these cases, VPAC2 immunoreactivity was uniformly present at the level of the plasma membrane of the majority (≥80%) of tumour cells (Fig. 3). In addition, a strong VPAC2 staining of tumoural blood vessels was noted, which markedly exceeded that of normal vasculature.

## Discussion

The VPAC2 receptor is involved in the regulation of normal rhythmic activity of the circadian clock, basal energy expenditure, immune response as well as male reproductive functions (1, 3, 32, 33, 34, 35, 36, 37). In addition, it has been shown that copy number variation leading to duplications of the *VIPR2* gene contributes to a significant risk for schizophrenia (3, 38). In an effort to study the pattern of VPAC2 receptor protein expression in normal and neoplastic human tissues, we extensively characterised the novel rabbit MAB SP235. We show that the cytoplasmic tail of the human VPAC2 receptor can serve as an epitope for the generation of antibodies that effectively stain formalin-fixed, paraffin-embedded mouse, rat and human tissues. Several lines of evidence indicate that SP235 specifically detects its targeted receptor and does not cross react. First, in Western blotting analysis of receptor-expressing cells, SP235 detected a broad band migrating at 50–70 kDa only in cells transfected with the VPAC2 receptor but not in cells transfected with the VPAC1 receptor or in untransfected cells. Second, SP235 revealed a prominent cell surface staining only in VPAC2-expressing cells, but not in VPAC1-expressing cells. Third, in the Western blots of a variety of mouse tissues, SP235 detected bands migrating at the appropriate molecular weight. Fourth, tissue immunostaining of SP235 was completely abolished by preadsorbtion with homologous but not heterologous peptides.

SP235 yielded an effective immunohistochemical staining of formalin-fixed, paraffin-embedded tissues with a predominance of plasma membrane staining and very low cytoplasmic signal. The use of SP235 also permitted us to gain novel insights into VPAC2 receptor expression and function. Prominent VPAC2 immunoreactivity was not only observed in blood vessels but also in gastric mucosa, exocrine pancreas and kidney tubules. In addition, previously unappreciated cellular localisations of VPAC2 receptors were uncovered, i.e. the presence of VPAC2 receptors at the plasma membrane of immune cells in all major lymphoid organs.

The rabbit MAB SP235 will also overcome a number of limitations inherent to polyclonal VPAC2 antibodies. Naturally, only limited amounts of polyclonal antibodies exist, and the quality of these antibodies varies from batch to batch (39). To achieve high-quality labelling affinity, purification is often required which limits their availability even further (39). In contrast, the rabbit MAB SP235 can be produced in unlimited amounts for an unlimited time in identical high quality. Thus, the development of SP235 will facilitate the elucidation of the precise tissue distribution, regulation and function of VPAC2 receptor in mouse, rat and human tissues.

VIP and PACAP can affect the growth of human tumour cells *in vivo* and *in vitro* (8, 11, 40, 41, 42). Whereas growth-promoting activities have been reported for VIP, growth-inhibiting properties have been found for VIP antagonists in various tumour models (11, 43). VPAC2 receptors are detectable in some of the most frequently occurring human tumours, including gastric cancer, pancreatic ductal adenocarcinoma and small-cell lung carcinoma (4, 5, 6, 7, 8, 9, 10, 11, 12, 13, 14). In addition, VPAC2 receptors exhibited a particular high prevalence in endocrine and neuroendocrine tumours such as thyroid carcinomas, GH-producing adenomas and neuroendocrine tumours of the gastrointestinal tract (4, 5, 6, 7, 8, 9, 10, 11, 12, 13, 14). It is therefore very tempting to suggest the use of growth-inhibiting VIP/PACAP analogues for the treatment of human tumours. Moreover, a number of clinical and preclinical studies suggest that VPAC2 receptors may be promising molecular targets for tumour imaging and targeted radiotherapy (44, 45). However, previous and present evidence indicates a highly abundant expression of VPAC2 receptors in many normal tissues but not in the heart. Thus, for potential clinical application of VIP/PACAP analogues, the ratio of VPAC2 receptor expression in neoplastic vs normal tissues should be determined using SP235 immunohistochemistry.

In a previous study, VIP/PACAP receptor binding sites have been detected using ^125^I-VIP. However, this method cannot easily distinguish between receptor subtypes and may thus lead to over- or underestimation of either VPAC1 or VPAC2 expression, in particular in tissues co-expressing these receptors (40).

In conclusion, we have generated and extensively characterised the novel rabbit monoclonal anti-VPAC2 antibody SP235. It is now possible to determine the exact cellular and subcellular sites of VPAC2 receptor protein expression in normal human tissues, which is needed for a better understanding of VIP/PACAP actions in physiological target tissues. The rapid immunocytochemical VPAC2 receptor visualisation using SP235 may also be helpful to identify candidate tumours for VIP-based diagnostics or therapeutic interventions.

## Supplementary data

This is linked to the online version of the paper at http://dx.doi.org/10.1530/EC-14-0051.

## Figures and Tables

**Table 1:** Prevalence of VPAC2 receptors in selected human tumours.

**Tumour type** (*n*)	**VPAC2**^a^ (*n*)
Gastric cancer (20)	12
Gastrointestinal stromal tumours (4)	2
Pancreatic ductal adenocarcinoma (10)	6
Small-cell lung carcinoma (5)	4
Lung adenocarcinoma (5)	1
Squamous cell carcinoma of the lung (46)	2
Sarcoma (19)	13
Pleomorphic sarcoma (2)	2
Leiomyosarcoma (4)	1
Rhabdomyosarcoma (46)	3
Liposarcoma (4)	2
Angiosarcoma (2)	2
Osteosarcoma (4)	3
Thyroid carcinoma (2)	2
Breast cancer (10)	4
Ovarian carcinoma (12)	3
Cervical carcinoma (13)	4
Urinary bladder carcinoma (8)	0
Prostate cancer (7)	0
Neuroendocrine tumours (23)	5
Pituitary adenoma, growth-hormone-producing (21)	10
Pheochromocytoma (5)	0

a A tumour sample was considered positive if at least 30% of tumour cells exhibited a moderate-to-strong staining of the plasma membrane.
